# Профилактика йододефицитных заболеваний: в фокусе региональные целевые программы

**DOI:** 10.14341/probl13119

**Published:** 2022-04-27

**Authors:** И. И. Дедов, Н. М. Платонова, Е. А. Трошина, Н. П. Маколина, И. М. Беловалова, Е. С. Сенюшкина, Г. А. Мельниченко

**Affiliations:** Национальный медицинский исследовательский центр эндокринологии Министерства здравоохранения Российской Федерации; Национальный медицинский исследовательский центр эндокринологии Министерства здравоохранения Российской Федерации; Национальный медицинский исследовательский центр эндокринологии Министерства здравоохранения Российской Федерации; Национальный медицинский исследовательский центр эндокринологии Министерства здравоохранения Российской Федерации; Национальный медицинский исследовательский центр эндокринологии Министерства здравоохранения Российской Федерации; Национальный медицинский исследовательский центр эндокринологии Министерства здравоохранения Российской Федерации; Национальный медицинский исследовательский центр эндокринологии Министерства здравоохранения Российской Федерации

**Keywords:** йодный дефицит, йодированная соль, йододефицитные заболевания, региональная программа профилактики

## Abstract

В основе стратегии ликвидации заболеваний, связанных с дефицитом йода, в масштабах Российской Федерации лежит принятие федерального закона, предусматривающего использование йодированной соли в качестве средства массовой (популяционной) йодной профилактики. Хронический дефицит йода, существующий в России, приводит к драматическим последствиям: развитию умственной и физической отсталости детей, кретинизму, заболеваниям щитовидной железы, бесплодию. В условиях йодного дефицита в сотни раз возрастает и риск радиационно-индуцированного рака щитовидной железы у детей в случае ядерных катастроф. По определению, все йододефицитные заболевания (ЙДЗ) могут быть предотвращены, тогда как изменения, вызванные нехваткой йода на этапе внутриутробного развития и в раннем детском возрасте, являются необратимыми и практически не поддаются лечению и реабилитации. Фактическое среднее потребление йода жителем России составляет всего 40–80 мкг в день, что в 3 раза меньше установленной нормы (150–250 мкг). Ежегодно в медицинские учреждения обращаются более 1,5 млн взрослых и 650 тыс. детей с различными заболеваниями щитовидной железы. Причиной 65% случаев заболеваний щитовидной железы у взрослых и 95% случаев у детей является недостаточное поступление йода с питанием. На этапе подготовки соответствующего законодательного акта разработка и реализация региональных программ профилактики ЙДЗ имеют исключительно важное значение. Типовой проект такой программы предложен в данной статье для ее адаптации и использования на региональном уровне.

## Abstract (en)

The strategy for the elimination of diseases associated with iodine deficiency throughout the Russian Federation is based on the adoption of a federal law providing for the use of iodized salt as a means of mass (population) iodine prophylaxis. Chronic iodine deficiency that exists in Russia leads to dramatic consequences: the development of mental and physical retardation in children, cretinism, thyroid diseases, and infertility. Under conditions of iodine deficiency, the risk of radiation-induced thyroid cancer in children in the event of nuclear disasters increases hundreds of times. By definition, all iodine deficiency diseases (IDDs) can be prevented, while changes caused by iodine deficiency during fetal development and in early childhood are irreversible and practically defy treatment and rehabilitation. The actual average consumption of iodine by a resident of Russia is only 40–80 mcg per day, which is 3 times less than the established norm (150–250 mcg). Every year, more than 1.5 million adults and 650 thousand children with various thyroid diseases turn to medical institutions. The cause of 65% of cases of thyroid disease in adults and 95% in children is insufficient intake of iodine from the diet. At the stage of preparing the relevant legislative act, the development and implementation of regional programs for the prevention of IDD is of utmost importance. A typical draft of such a program is proposed in this article for its adaptation and use at the regional level.

## РАЗДЕЛ 1

На протяжении последних 20 лет в России предпринимаются попытки осуществления законодательного регулирования йодной профилактики. Соответствующие законопроекты разрабатывались Минздравом России, дорабатывались с учетом замечаний и предложений Федеральных органов исполнительной власти (ФОИВ), однако на этапах оценки регулирующего воздействия не находили поддержки. Так произошло и с Проектом Федерального закона (ФЗ) «О профилактике заболеваний, вызванных дефицитом йода», разработанным Министерством здравоохранения Российской Федерации во исполнение пункта 50 плана мероприятий по реализации Стратегии повышения качества пищевой продукции в Российской Федерации до 2030 г., утвержденной распоряжением Правительства Российской Федерации от 19 апреля 2017 г. № 738-р, подпункта «а» пункта 1 перечня поручений Президента Российской Федерации В.В. Путина от 3 июля 2018 г. № Пр-1136, поручения заместителя Председателя Правительства Российской Федерации Т.А. Голиковой от 15 июня 2018 г. № ТТ-П12-3408.

Основная аргументация при оценке регулирующего воздействия (ОРВ) — законопроект «содержит положения, вводящие избыточные обязанности, запреты и ограничения для физических и юридических лиц в сфере предпринимательской и иной экономической деятельности или способствующие их введению, а также положения, приводящие к возникновению необоснованных расходов физических и юридических лиц в сфере предпринимательской и иной экономической деятельности, а также бюджетов всех уровней бюджетной системы РФ» (№ 10890-АХ/Д26и от 09.04.2021, Минэкономразвития РФ, «Заключение об оценке регулирующего воздействия на проект Федерального закона “О профилактике заболеваний, вызванных дефицитом йода”») [1].

Несмотря на высокую распространенность йододефицитных заболеваний (ЙДЗ), на успешный опыт многих стран мирового сообщества, решивших проблему путем популяционной профилактики с использованием йодированной соли, «угроза бизнесу» в России, где люди продолжают испытывать хронический дефицит йода с развитием всего спектра указанных выше патологий, представляется более серьезной, чем угроза здоровью, когда речь идет о простом, доступном, дешевом и доказанно эффективном способе йодной профилактики — использовании йодированной соли.

## РАЗДЕЛ 2

Идет время, распространенность и заболеваемость ЙДЗ, особенно в группах риска — у детей и беременных женщин, растут.

Статистика заболеваемости отмечает не только лидирующие позиции заболеваний щитовидной железы (ЩЖ) в структуре всей эндокринной патологии, составляющие 27,6% по данным Росстата за 2020 г., ведущее место суммарной доли зоба (эндемический + нетоксический) и синдрома врожденной йодной недостаточности среди всей патологии ЩЖ (достигает 49%), но и неумолимую динамику роста заболеваемости ЩЖ у жителей Российской Федерации с увеличением вдвое за последние 10 лет. Очевидно, что это связано в первую очередь с хроническим дефицитом йода в питании населения страны, причем, по данным Росстата:

## РАЗДЕЛ 3

Нужно отметить, что за последние 20 лет в России неоднократно предпринимались попытки решить вопрос профилактики йодного дефицита на региональном уровне, ярким примером этого был период 2000–2005 гг., когда в 43 субъектах России были приняты и реализованы региональные программы профилактики ЙДЗ. Однако проведенный по поручению Минздрава России анализ этих программ показал, что большинство из них было ориентировано на приобретение оборудования для обследования населения, а также на внедрение пищевых и биологически активных добавок, содержащих йод, в качестве средств йодной профилактики. Цель этих программ достигнута не была, основные биологические индикаторы йодной обеспеченности населения практически не изменились, распространенность ЙДЗ осталась прежней. Все данные опубликованы [3–6]. Несостоятельность такого подхода к массовой йодной профилактике вкупе с финансовыми затратами на обогащение продуктов питания йодом, существенно превышающими таковые на йодирование соли на этапе ее производства, были учтены при разработке Минздравом России законопроекта «О профилактике заболеваний, вызванных дефицитом йода».

За прошедшие 20 лет ФГБУ «НМИЦ эндокринологии» по поручению Минздрава России провел серию контрольно-эпидемиологических исследований в регионах страны. Полученные данные опубликованы и убедительно свидетельствуют об эффективности использования йодированной соли для популяционной йодной профилактики, ярким примером этого может служить динамика обеспеченности йодом, заболеваемости зобом и синдромом врожденной йодной недостаточности (кретинизмом) в республике Тыва (регион тяжелого природного йодного дефицита). За 10 лет внедрения популяционной йодной профилактики йодированной солью дефицит йода в питании населения республики устранен, а по уровню распространенности йододефицитного зоба Тыва стала сопоставима с южными приморскими регионами России, снизив его за истекшее десятилетие в 10 раз [3–9].

## РАЗДЕЛ 4

Исключительная важность законодательного регулирования йодной профилактики в России связана с тем, что до настоящего времени профилактические мероприятия в стране не носили постоянного и систематического характера, не охватывали все население, а средства для профилактики нередко не соответствовали международным стандартам.

В Российской Федерации йодированную соль в питании употребляют менее 30% населения в отличие от входящих в Таможенный союз ЕврАзЭС стран, где введено йодирование соли и на фоне этого достигнут впечатляющий прогресс в устранении заболеваний, связанных с дефицитом йода. Убедительно доказано, что медицинских противопоказаний для использования йодированной соли в питании не существует, передозировка йода, поступающего с йодированной солью в организм человека, исключена. Доступность массовой (популяционной) йодной профилактики очевидна. Так, увеличение стоимости при производстве йодированной соли не превышает 2 рубля на человека в год. Для соляной промышленности России не существует реальных препятствий для полного обеспечения потребности страны в йодированной соли.

Следует отметить важность принимаемых мер, направленных на профилактику ЙДЗ со стороны Роспотребнадзора. Так, изменения, внесенные в СанПиН 2.3/2.4.3590-20 «Санитарно-эпидемиологические требования к организации общественного питания населения», позволили обеспечить обязательное использование йодированной соли в организованном питании детей. Это очень серьезный шаг в профилактике ЙДЗ. Логично, что объединение усилий двух ключевых ведомств, ответственных за здоровье граждан страны, Минздрава России и Роспотребнадзора, в доработке и продвижении законопроекта «О профилактике заболеваний, вызванных дефицитом йода» было бы исключительно важным.

В отсутствие закона исключительно актуальным шагом на пути решения задачи по борьбе с ЙДЗ должно стать формирование единого в масштабах страны профилактического процесса, базирующегося на соответствующей нормативно-правовой базе в каждом субъекте РФ, — разработка и реализация целевых региональных программ по профилактике заболеваний, вызванных дефицитом йода.

Приказом Минздрава России от 15.01.2020 №8 утверждена «Стратегия формирования здорового образа жизни населения, профилактики и контроля неинфекционных заболеваний на период до 2025 года», для решения задач Стратегии в числе основных направлений указано:

Все вышеперечисленные направления Стратегии отражают ключевые вопросы системной йодной профилактики и полностью гармонизированы с ее необходимостью.

## РАЗДЕЛ 5

Эффективная реализация концепции единого профилактического пространства в контексте йодной профилактики возможна как в масштабах субъекта РФ, так и на уровне его отдельных муниципальных образований (городов, районов), за которыми в законодательном порядке формально закреплена возможность получения от органов исполнительной власти полномочий по осуществлению органами местного самоуправления деятельности, способствующей укреплению здоровья человека, экологическому и санитарно-эпидемиологическому благополучию. Наглядный пример такого подхода демонстрирует Республика Тыва, где йодная профилактика осуществляется на всех уровнях, начиная с муниципального (рис. 1).

**Figure:**
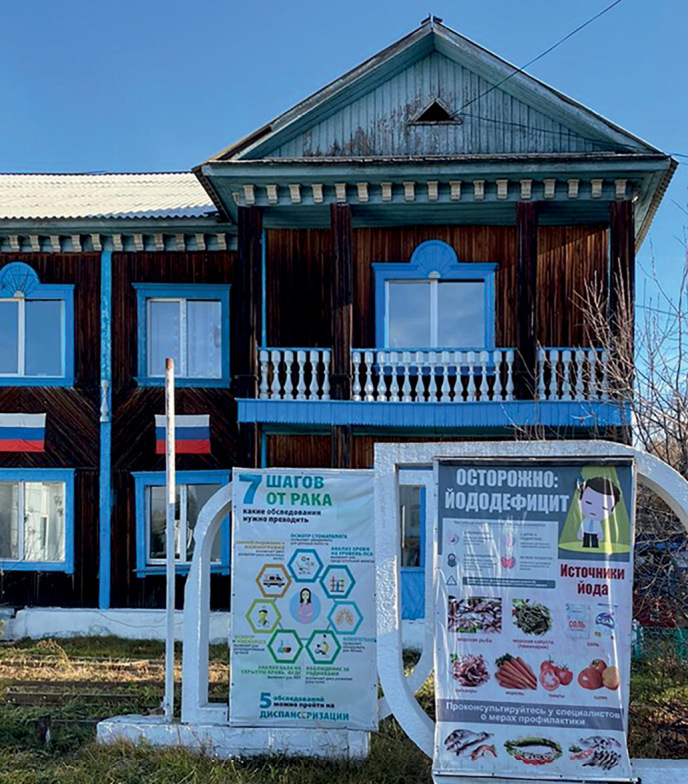
Рисунок 1. Плакат перед поликлиникой в районном центре Республики Тыва (2020 г.) — наглядный пример профилактической работы в регионе на уровне муниципального образования.

Формирование системы общественного здравоохранения, проводимое с учетом условий каждого конкретного региона страны, используя и развивая потенциал различных государственных отраслей и общественных организаций, а не только усилиями медицинских работников внутри сферы здравоохранения, приведет к значимо лучшим результатам в решении вопросов профилактики ЙДЗ.

В качестве одного из примеров межведомственного взаимодействия можно привести изданное в 2016 г. распоряжение Правительства Республики Тыва «Об утверждении межведомственного плана мероприятий по формированию здорового образа жизни у населения Республики Тыва на 2016–2018 годы», согласно которому предприятиям пищевой и перерабатывающей промышленности рекомендовано использовать йодированную соль при производстве молочной продукции и хлебобулочных изделий; управлению Роспотребнадзора и Министерству сельского хозяйства и продовольствия поручено проводить контрольные мероприятия по использованию йодированной соли при производстве продуктов питания. Данные мероприятия существенно повлияли на обеспеченность населения республики йодом [6][7].

Не исключено, что реализация этих мер стала причиной позитивных изменений, зафиксированных Росстатом, а именно: за период 2017–2019 гг. общая заболеваемость патологиями ЩЖ в Республике Тыва составила 3361,8 случая на 100 тыс. населения, в том числе на долю заболеваний, связанных с дефицитом йода, приходилось 2811,2 случая. По результатам неонатального скрининга заболеваемость врожденным гипотиреозом составила всего 2 случая на 6989 новорожденных в 2017 г., в 2018–2019 гг. случаев врожденного гипотиреоза зафиксировано не было [4]. В настоящее время основной акцент в регионе сделан на массовой йодной профилактике путем использования йодированной соли в питании населения.

## РАЗДЕЛ 6

Среди путей по осуществлению контроля над неинфекционными заболеваниями в аспекте ЙДЗ следует выделить прежде всего разработку комплекса мер различных уровней, синергия которых обеспечит эффективный результат выполнения целевой программы, включающего следующие направления:

Принципиально важно отметить, что для достижения успеха в борьбе с ЙДЗ и значимых результатов по ликвидации дефицита йода в питании населения в масштабах страны необходима консолидация усилий всех субъектов РФ, в которых будут разработаны профилактические программы с учетом особенностей и условий территории, а также ресурсов региона.

## РАЗДЕЛ 7

Целесообразной представляется унифицированная целевая программа, которая позволит охватить профилактическими мероприятиями все слои населения с использованием средств, соответствующих международным стандартам. Это, прежде всего, — массовая профилактика ЙДЗ при помощи йодированной соли на постоянной основе, закрепленной административными решениями (нормативно-правовыми документами), с контролем ее качества и индивидуальная йодная профилактика лекарственными препаратами йодида калия в группах высокого риска развития ЙДЗ (дети до 2 лет, беременные и кормящие женщины).

Мониторинг программы по устранению ЙДЗ должен включать исследование охвата йодной профилактикой населения (доля семей, употребляющих йодированную соль, в процентах) и медианы экскреции йода с мочой у детей препубертатного возраста, доли (%) новорожденных с уровнем ТТГ выше 5 мкМЕ/л (по данным неонатального скрининга) —не реже 1 раза в 2–3 года. Необходимо систематически осуществлять контроль эффективности профилактики заболеваний, связанных с дефицитом йода, с использованием целевых индикаторов: распространенности и заболеваемости диффузными и узловыми формами зоба, осложнений, вызванных ЙДЗ, количества новых технологий профилактики, диагностики и лечения ЙДЗ и т.д.

Мониторинг проводимых профилактических программ должен проводиться интегрированно с вовлечением таких организаций, как Министерство здравоохранения Российской Федерации (непосредственно или посредством курации профильным федеральным центром — ФГБУ «НМИЦ эндокринологии» Минздрава России) и Роспотребнадзора.

По оценке экспертов ФГБУ «НМИЦ эндокринологии» Минздрава России, реализация программы профилактики ЙДЗ в субъектах РФ приведет к существенному улучшению здоровья населения, а на каждый 1 рубль, вложенный в программу массовой профилактики ЙДЗ, будет получено 9 рублей в виде прироста производства уже в течение первых 3 лет функционирования программы. Это только поддающийся расчетам ожидаемый экономический эффект от повышения производительности труда, не считая социально-политического и морального эффекта, а также снижения затрат на лечение и реабилитацию.

Предложен проект типовой региональной целевой программы по профилактике йододефицитных заболеваний на уровне отдельного субъекта РФ [10].

## ЗАКЛЮЧЕНИЕ

Обеспечение эффективного результата выполнения целевой программы по профилактике ЙДЗ возможно только при разработке комплекса мер различных уровней, основой которых в итоге станет принятие федерального закона, предусматривающего использование йодированной соли в качестве средства массовой (популяционной) йодной профилактики.

## ДОПОЛНИТЕЛЬНАЯ ИНФОРМАЦИЯ

Источники финансирования. Работа выполнена в рамках государственного задания Минздрава России: «Эпидемиологические и молекулярно-клеточные характеристики опухолевых, аутоиммунных и йододефицитных тиреопатий как основа профилактики осложнений и персонализации лечения», Рег. № АААА-А20-120011790180-4.

Конфликт интересов. Авторы декларируют отсутствие явных и потенциальных конфликтов интересов, связанных с публикацией настоящей статьи.

Участие авторов. Дедов И.И., Трошина Е.А., Платонова Н.М., Беловалова И.М., Мельниченко Г.А. — концепция и дизайн исследования; Маколина Н.П., Сенюшкина Е.С. — обработка материалов, анализ полученных данных, написание текста. Все авторы одобрили финальную версию статьи перед публикацией, выразили согласие нести ответственность за все аспекты работы, подразумевающую надлежащее изучение и решение вопросов, связанных с точностью или добросовестностью любой части работы.
